# Increase in Mitochondrial D-Loop Region Methylation Levels in Mild Cognitive Impairment Individuals

**DOI:** 10.3390/ijms23105393

**Published:** 2022-05-12

**Authors:** Andrea Stoccoro, Filippo Baldacci, Roberto Ceravolo, Linda Giampietri, Gloria Tognoni, Gabriele Siciliano, Lucia Migliore, Fabio Coppedè

**Affiliations:** 1 Department of Translational Research and of New Surgical and Medical Technologies, University of Pisa, Via Roma 55, 56126 Pisa, Italy; lucia.migliore@unipi.it; 2 Department of Clinical and Experimental Medicine, University of Pisa, 56126 Pisa, Italy; filippo.baldacci@unipi.it (F.B.); roberto.ceravolo@unipi.it (R.C.); lindagiampietri@gmail.com (L.G.); gloria.tognoni@med.unipi.it (G.T.); gabriele.siciliano@unipi.it (G.S.)

**Keywords:** Alzheimer’s disease, mild cognitive impairment, DNA methylation, mitoepigenetics, mitochondrial D-loop region

## Abstract

Methylation levels of the mitochondrial displacement loop (D-loop) region have been reported to be altered in the brain and blood of Alzheimer’s disease (AD) patients. Moreover, a dynamic D-loop methylation pattern was observed in the brain of transgenic AD mice along with disease progression. However, investigations on the blood cells of AD patients in the prodromal phases of the disease have not been performed so far. The aim of this study was to analyze D-loop methylation levels by means of the MS-HRM technique in the peripheral blood cells of 14 mild cognitive impairment (MCI) patients, 18 early stage AD patients, 70 advanced stage AD patients, and 105 healthy control subjects. We found higher D-loop methylation levels in MCI patients than in control subjects and AD patients. Moreover, higher D-loop methylation levels were observed in control subjects than in AD patients in advanced stages of the disease, but not in those at early stages. The present pilot study shows that peripheral D-loop methylation levels differ in patients at different stages of AD pathology, suggesting that further studies deserve to be performed in order to validate the usefulness of D-loop methylation analysis as a peripheral biomarker for the early detection of AD.

## 1. Introduction

Alzheimer’s disease (AD), the most prevalent neurodegenerative disorder and the most common cause of dementia, is characterized by brain atrophy, extraneuronal deposition of amyloid-β (Aβ) plaques, and intraneuronal accumulation of neurofibrillary tangles [1]. A long preclinical phase precedes the onset of clinical symptoms by at least 10–20 years, and full-blown AD is usually preceded by a prodromal stage known as mild cognitive impairment (MCI). MCI is characterized by a milder degree of cognitive decline, not normal for subjects’ age and education, that does not cause a significant impairment of occupational or social functioning [2,3]. It is noteworthy that not every MCI subject will develop dementia eventually, with annual conversion rates varying from 5 to 10% according to different settings [4]. Among MCI subjects, amnesic single and multidomain impairment profiles, and the presence of cerebral beta-amyloidosis, particularly if coupled with positive neurodegeneration biomarkers, are associated with a higher conversion rate to overt AD dementia [5,6,7,8]. The clinical course of AD is characterized by the onset of severe and progressive disability, including memory dysfunction, and problems linked with language processing, orientation, critical thinking, and judgment, and with death generally within 5–12 years of symptoms onset [9]. Identifying patients in the early stages of the disease could greatly improve clinical management, providing a promising window into the disease progression for therapeutic interventions capable of preventing or slowing down the course of the disease, which, unfortunately, are not yet available. To date, the use of biological markers derived from cerebrospinal fluid, including Aβ1-42 peptide, total tau (t-tau), and phosphorylated-tau (p-tau) proteins, and neuroimaging analysis, including amyloid positron emission tomography (PET) imaging to measure the amount of protein deposit in the brain and magnetic resonance imaging (MRI) measurements of brain volume and neuronal connectivity, together with neurological examinations, is the most accurate approach to achieve the diagnostic sensitivity and specificity necessary to identify people in the earliest stages of the disease [10]. However, analyses to identify such biological markers are invasive for the patients and expensive for the healthcare system, so the ideal AD biomarker panel will likely involve blood biomarkers associated with AD pathophysiologic mechanisms and provide insights into the onset and development of the disease.

Pathogenetic mutations in three genes, namely beta-amyloid precursor protein (*APP*), presenilin 1 (*PSEN1*), and presenilin 2 (*PSEN2*), which induce an overproduction of the Aβ peptide, cause an early-onset (before age 65) autosomal dominant familial inherited form of the disease, representing a small percentage (<1%) of AD cases [11]. The sporadic late-onset (onset after 65 years of age) form of AD, which accounts for the majority of AD cases, is likely due to the interaction of several genetic factors, with apolipoprotein E ε4 (*APOEε4*) as the main genetic risk factor, and environmental factors, including brain injury, diabetes, cardiovascular disease, and exposure to various pollutants, and especially increasing age [12]. Although the exact etiology of sporadic AD is not well understood, several pieces of evidence indicate a pivotal role played by mitochondrial dysfunction, which has been suggested as the *primum movens* for the further development of the molecular alterations that characterize the disease, such as impaired apoptosis, disruption of calcium homeostasis, inflammation, oxidative stress, and deficient glucose metabolism, ultimately leading to neuronal death [13]. In line with this, the “mitochondrial cascade hypothesis” suggests that mitochondrial dysfunction is an early and primary essential event in the pathogenesis of AD [14]. This hypothesis is based on evidence from several studies performed in the brain and peripheral tissues from animal models of AD and human patients, which show altered mitochondrial metabolism even before detectable changes in Aβ [15]. Furthermore, the localization of dysfunctional mitochondria in non-degenerative and non-neuronal sites, but also outside the brain, indicates that they are not exclusive byproducts of neurodegeneration [16].

In recent years, numerous evidences have revealed the fundamental role played by the epigenetic mechanisms in the etiology of AD [17]. Epigenetic mechanisms, such as DNA methylation and histone tail modifications, can influence gene expression, and thus the etiopathogenesis underlying several human complex diseases, including cancer, metabolic disorders, and cardiovascular and neurodegenerative diseases [18]. Interestingly, an increasing number of studies have shown that mitochondrial DNA (mtDNA) replication and gene expression are also regulated by epigenetic mechanisms (mitoepigenetics), and their alteration has been suggested to underlie several pathological pathways, including neurodegeneration [19,20]. The main mtDNA region investigated in mitoepigenetic studies is the displacement loop (D-loop) region, an mtDNA sequence involved in the regulation of both mtDNA replication and transcription [21]. The alteration in the D-loop methylation pattern has been observed in the spinal cord and skeletal muscle cells of human-*SOD1* transgenic amyotrophic lateral sclerosis (ALS) mice [22] and in the peripheral blood of patients with both familial and sporadic forms of ALS [23,24]. Altered levels of D-loop methylation have also been detected in brain samples from patients with Parkinson’s disease (PD) [25] but not in their peripheral blood [26,27]. Regarding AD, animal studies revealed that D-loop methylation levels were altered in brain samples of APP/PS1 transgenic mice and changed during disease progression [25,28]. Investigations of human samples revealed different D-loop methylation patterns in both post-mortem and peripheral blood samples from AD patients compared to neurologically healthy controls [25,29]. In particular, the levels of D-loop methylation were found to be altered in the entorhinal cortex of eight patients with AD-related pathology, and were especially higher in patients in the early stages of the disease than in patients in the advanced stages [25]. On the other hand, we observed a 25% reduction in D-loop methylation levels in the peripheral blood of late-onset AD patients in the advanced stages of the disease compared to age and sex-matched healthy controls [29]. Taken together, human and animal studies performed to date suggest that D-loop methylation may be involved in the pathogenesis of AD, providing new information for understanding the biology of the disease and potentially providing peripheral biomarkers of the disease. However, as far as we know, until now D-loop methylation levels have not been investigated in the peripheral blood of patients with AD at different stages of the disease.

To further address the involvement of mtDNA methylation in the pathogenesis of AD and to detect potential peripheral biomarkers of AD, in the present study we assessed the levels of D-loop methylation in the peripheral blood of MCI patients and AD patients at both early and advanced stages of the disease.

## 2. Results

### 2.1. MS-HRM D-Loop Methylation Analyses

In the present study, methylation levels of the mitochondrial D-loop region were investigated by methylation-sensitive high resolution melting (MS-HRM) in 102 patients in the spectrum of AD, who, according to their clinical dementia rating (CDR), were classified as MCI (CDR = 0.5; *n* = 14), AD at early stages of the disease (CDR = 1, *n* = 18), and AD at advanced stages of the disease (CDR ≥ 2; *n* = 70), as well as in 105 neurologically healthy control subjects matched for age and sex with patients. MS-HRM experiments showed the existence of high interindividual variability with D-loop methylation levels ranging from 0% to approximately 12%. Table 1 shows the mean D-loop methylation levels obtained in control subjects, MCI patients, and patients with early and advanced AD.

### 2.2. Effects of Age and Sex on D-Loop Methylation Levels

**Figure 1:**
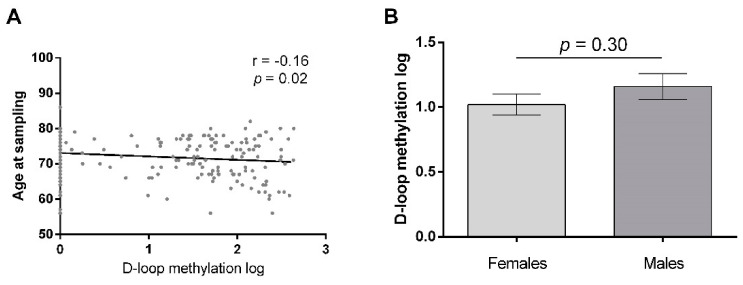
Effect of age at sampling (**A**) and sex (**B**) on natural logarithm D-loop methylation levels (derived by natural logarithm transformation of MS-HRM D-loop methylation percentage). Pearson correlation coefficient was used to evaluate the correlation between D-loop methylation levels and age at sampling. The difference in D-loop methylation between females and males was calculated by ANOVA; data are expressed as mean ± SEM.

In order to investigate if age and sex should be included as confounding factors in our statistical analyses, we investigated if they had an effect on D-loop methylation levels. Pearson correlation revealed that D-loop methylation levels were inversely correlated with age at sampling (r = −0.16; *p* = 0.02, Figure 1a). One-way ANOVA showed that there was not a statistically significant difference in D-loop methylation levels between females and males (*p* = 0.30, Figure 1b). In accordance with these results, we included only age as a covariate in the subsequent statistical analyses.

### 2.3. D-Loop Methylation in Different Diagnostic Groups

**Figure 2:**
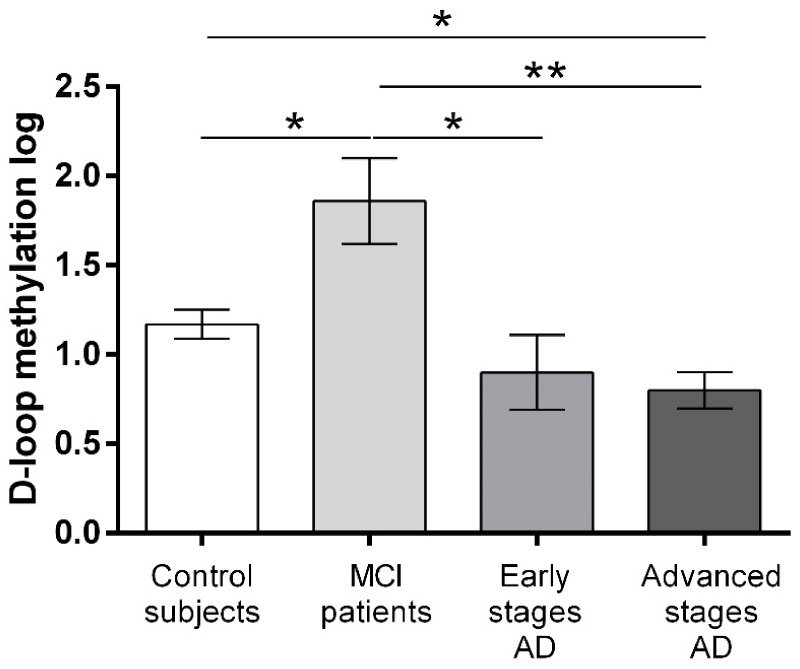
MS-HRM natural logarithm D-loop methylation levels (derived by natural logarithm transformation of D-loop methylation percentage) in the four groups of subjects. Statistical analysis was performed by means of ANCOVA, including sex and age at sampling as covariates. Data are expressed as mean ± SEM. Only *p*-values that survived correction for multiple comparisons are shown. * *p* < 0.05; ** *p* < 0.01.

The analysis of the covariance, corrected for age at sampling, showed the presence of statistically significant differences in D-loop methylation levels among the groups analysed (Figure 2). Notably, D-loop methylation levels were higher in MCI patients than in control subjects (*p* = 0.041), early stage AD patients (*p* = 0.019), and advanced stage AD patients (*p* = 0.0006). Control subjects had higher D-loop methylation levels than AD patients in the advanced disease stages (*p* = 0.04), but were not different compared to AD patients in early AD stages (*p* = 0.94). There was no significant difference between early stage AD and advanced stage AD patients (*p* = 0.99).

### 2.4. Prediction of MCI Status Using D-Loop Methylation Levels

**Figure 3:**
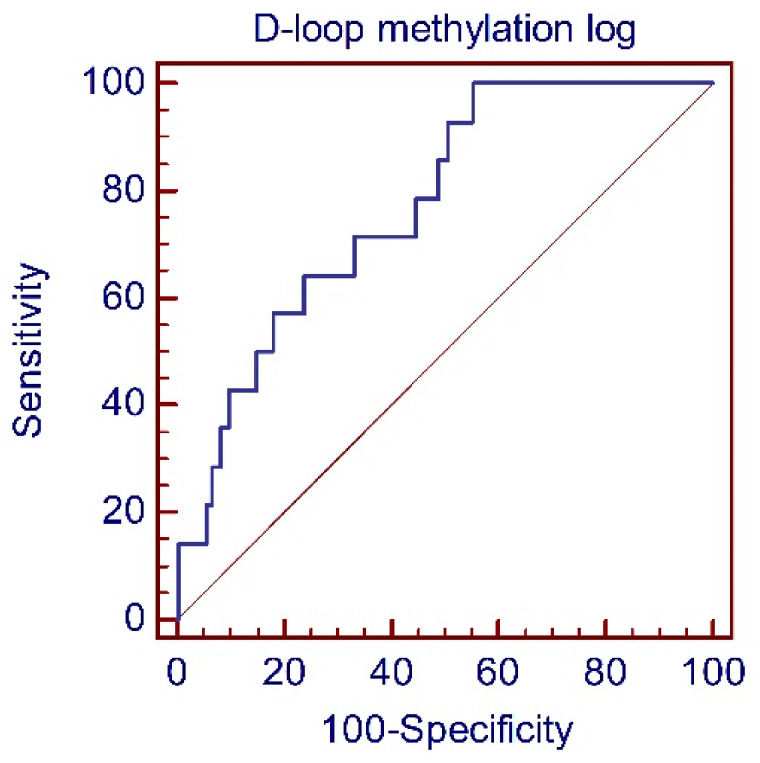
Receiver operating characteristic curve using D-loop methylation data. MCI patients were compared with the other groups. AUC = 0.771 (95% CI: 0.707–0.826; *p* = 0.0001). AUC: area under the curve.

Given that MCI patients showed the most altered methylation levels compared to the other groups of individuals, we assessed the accuracy of D-loop methylation for the detection of MCI condition by means of receiver operating characteristic (ROC) analysis and estimating the area under the curve (AUC). The analysis (Figure 3) revealed an acceptable capacity for discriminating between MCI patients and the other individuals, including control subjects and early and advanced stage AD patients with an AUC = 0.771 [95% CI: 0.707–0.826; *p* = 0.0001].

### 2.5. Correlations between D-Loop Methylation and Clinical and Biochemical Parameters

We then investigated the presence of associations between D-loop methylation levels and clinical (mini-mental state examination, MMSE) and biochemical (CSF t-tau, p-tau, and Aβ1-42) parameters available for the MCI and early AD patients. Partial correlations controlling for age (Table 2) showed a significant negative correlation between D-loop methylation levels and CSF p-tau (r = −0.64; *p* = 0.025), and a negative correlation with a tendency towards significance between D-loop methylation and CSF t-tau (r = −0.53; *p* = 0.062). No significant correlations were detected between D-loop methylation levels and MMSE (r = 0.05; *p* = 0.76) and Aβ42 levels (r = −0.39; *p* = 0.18).

## 3. Discussion

There is a growing interest in the study of mitoepigenetic mechanisms in relation to human health and pathology. Given the pivotal role that mitochondria play in neurodegeneration, mitoepigenetic investigations could provide new insights into the mechanisms underlying mitochondrial dysfunction in neurodegenerative diseases, as well as provide new disease biomarkers. The aim of the present study was to broaden our knowledge on the involvement of mtDNA methylation alteration in AD. To this end, we investigated the methylation levels of the regulatory D-loop region of the mitochondrial genome in the peripheral blood of 14 patients with MCI, 18 patients with AD in the early stages of the disease, 70 patients with AD in the advanced stages of the disease, and of 105 healthy control subjects. We observed that MCI patients had increased D-loop methylation levels compared to control subjects and to AD patients both in the early and advanced stages of the disease. In addition, control subjects showed increased D-loop methylation levels compared with AD patients in the advanced stages of the disease but not with patients with early AD. ROC analysis confirmed the capability of D-loop methylation for discriminating MCI patients from the other individuals. We also observed that D-loop methylation was inversely related to CSF p-tau levels and age at sampling.

The study of mitoepigenetic alterations in AD has been little investigated, but the results obtained so far suggest that this research field deserves to be further explored. In the first “mitoepigenetic” investigation of AD, a non-significant increase in mtDNA global hydroxy-methylation levels was found in post-mortem brain tissues of seven late-onset AD patients compared to five control subjects [30]. Quantification of DNA methylation at single-base resolution showed increased D-loop and decreased *MT-ND1* gene methylation levels in the entorhinal cortex of AD patients with early-stage AD-related pathology compared to brain samples from control subjects, as well as a dynamic D-loop methylation pattern in the neocortex of APP/PSEN1 mice along with the progression of AD pathology [25]. Subsequently, we observed a decrease in the levels of methylation of D-loop in the peripheral blood of patients with AD compared to control subjects [29]. In two studies performed on hippocampus specimens from AD and wild-type mice, a research team found a decrease in D-loop methylation levels and an increase in methylation of *mt-Rnr1*, *Cytb,* and *Cox-2* mitochondrial genes with a concurrent reduction in the mtDNA copy number and gene expression in the hippocampus of AD mice [28,31]. The findings of the present study add new knowledge to the field of mitoepigenetics in AD and suggest that peripheral blood D-loop methylation levels could be sensitive to different stages of AD progression.

The observation by Blanch et al. [25] that D-loop methylation changed during disease progression in a mouse model of AD, suggested that methylation levels of this mitochondrial region may be sensitive to different stages of the disease. Specifically, when comparing D-loop methylation levels of the cerebral cortex of control mice and of AD mice at different ages, authors observed that D-loop methylation levels were lower in AD mice at 3 months of age, higher at 6 months of age and again, and most strongly, lower in mice at 12 months of age. Furthermore, in the same study, higher D-loop methylation levels were observed in the entorhinal cortex of patients with AD-related pathology with Braak stages I to II compared to AD-related pathology cases with Braak stages from III to IV. Considering that Braak I/II stages correspond to an initial clinically silent phase of AD, combining the results of Blanch and collaborators with the results of the present study, it should be assumed that D-loop methylation levels are modulated during the progression of AD and that this modulation is identifiable both in the central nervous system and in the peripheral blood. D-loop methylation changes are likely linked to the mitochondrial metabolism that characterizes AD pathogenesis, already in the prodromal stages of the disease, by modulating mtDNA replication and gene expression [32]. Altered mtDNA replication has been frequently observed in both brain tissues and peripheral blood of AD patients, although it is still difficult to determine how mtDNA replication changes in the tissues of patients with AD during disease progression [33,34]. In a study performed on the peripheral blood of control subjects, and patients with MCI and AD of Italian and Spanish origins, an overall increase in the number of copies of mtDNA was found in control subjects compared to both patients with MCI and AD [34]. Regarding the differences between MCI and AD patients, the authors observed an increase in mtDNA copy number in AD patients compared to MCI patients in the Italian cohort, while an opposite trend was observed in the Spanish cohort [34]. On the other hand, a clearer picture is known regarding changes in gene expression during the pathogenesis of AD, which has often been found to be increased in MCI and decreased in AD patients, and several mechanisms, including epigenetic mechanisms, gene compensation and altered control of RNA production, have been proposed to explain this phenomenon [35]. Gene expression analysis in four cortical brain regions revealed that expression of genes associated with mitochondrial energy generation was prominently upregulated in MCI brains compared to control subjects, and to a greater extent to AD patients, suggesting that the MCI brain undergoes dramatic upregulation of energy metabolism genes, in contrast to the transcriptional downregulation associated with AD [36]. Similarly, a study performed in hippocampal tissues reported a significant downregulation in nucleus-encoded oxidative phosphorylation (OXPHOS) genes in AD patients as well as in the *MT-ND6* gene, while the same genes were upregulated in subjects with MCI [37]. In a subsequent study, Lunnon and collaborators observed a reduction in the expression levels of nuclear genes along with a concomitant increase in the expression of the OXPHOS genes encoded in mitochondria in the white blood cells of MCI and AD patients compared with controls, with the magnitude of major differential expression in individuals with AD [38]. In the study conducted by Blanch and collaborators described above, increased expression of the mitochondrial gene *MT-ND1* was detected in Braak stages V/VI patients compared to controls and to Braak stages I/II patients, and the authors suggested that the increased *MT-ND1* expression in the advanced stages of the disease were probably due to the decreased *MT-ND1* methylation levels observed in the early stages [25]. Overall, these studies showed that mitochondrial activity greatly changes among control, MCI, and AD subjects, reflecting different demands of the mitochondrial activity or different stages of mitochondrial dysfunction. It should be hypothesized that the increased D-loop methylation we observed in MCI patients reflects a compensatory mechanism for the sudden increase in mitochondrial activity observed in those patients, or maybe it is the initial driver of mitochondrial hypometabolism that characterizes AD. Further work aimed at investigating how D-loop methylation is linked to disease progression, and why its methylation levels increase in MCI to then decline as the disease progresses, needs to be performed. Unfortunately, in the present study, we had no RNA samples available to study how D-loop methylation levels affect the mtDNA gene expression in our cohort. Moreover, we did not have repeated samplings from the same patients at different disease stages, so we were unable to longitudinally address how D-loop methylation levels are dynamically regulated along with disease progression.

In recent years, epigenetic biomarkers have greatly improved the management of patients affected by different human diseases, particularly cancer, providing biomarkers, some of which are approved by the US Food and Drug Administration (FDA), for diagnosis, prognosis, or response to therapy, as well as for the development of epigenetic-based therapy [39]. Much effort is underway to identify epigenetic biomarkers of AD, and consistent evidence suggests the usefulness of blood DNA methylation as a source of peripheral biomarkers sensitive to AD progression. Indeed, peripheral blood methylation levels of several nuclear genes, including *CPT1B*, *APP*, *BDNF*, *NCAPH2/LMF2*, *PM20D1*, and *OXT*, have already been associated with the progression of AD [40,41,42,43,44,45,46,47]. Notably, increased *BDNF* promoter methylation levels were observed in MCI patients compared to controls and were particularly high in those patients who converted to AD compared to unconverted individuals at a 5-year follow-up [41]. Another gene associated with MCI to AD conversion was *PM20D1* [42,43]. Interestingly, from longitudinal data, promoter hypomethylation of *PM20D1* during MCI and the early AD stage has been shown to be reversed into promoter hypermethylation in the late AD stage [43]. Several differentially methylated regions (DMRs) were identified in an epigenome-wide association study in non-demented individuals who converted to AD dementia versus unconverted subjects [46]. Interestingly, one of these DMRs included CpG sites near the transcriptional start site of the *OXT* gene, which was also found to be altered in the middle temporal gyrus of AD patients, thus suggesting that the altered peripheral blood methylation levels could mirror the DNA methylation alterations of brain tissues [46]. Overall, these studies clearly suggest that peripheral DNA methylation could be sensitive to the progression of AD pathogenesis, and could provide peripheral biomarkers of the disease. Although the number of MCI patients who converted to AD in our cohort was too low (5 out of 14) to investigate whether D-loop methylation levels can predict the conversion from MCI to AD, the results of our study show that also peripheral blood D-loop methylation deserves to be considered as a potential biomarker for AD. Indeed, ROC analysis revealed an acceptable capability of D-loop methylation to discriminate between MCI and the other subjects recruited. Moreover, we observed that D-loop methylation inversely correlated with CSF p-tau levels, and, with a tendency toward significance, with CSF t-tau, but not with Aβ1-42. This is an interesting observation considering that, although CSF p-tau, t-tau, and Aβ1-42 are all necessary for AD diagnosis, constituting the core AD CSF biomarkers, only p-tau seems to be specific for AD, while altered t-tau and Aβ1-42 levels could be found also in CSF of patients affected by other neurodegenerative diseases and acute brain disorders [48]. Thus, this finding further suggests that peripheral D-loop methylation could be informative for AD pathophysiological mechanisms considering that CSF p-tau reflects the intensity of neurodegeneration and progression of the disease and is strictly related to AD-related mitochondrial dysfunction [49,50]. Future studies in a larger cohort of individuals with MCI followed for a long time are needed to confirm current results and to reveal if they can help to identify biomarkers of MCI to AD conversion.

The inverse correlation observed between age at sampling and D-loop methylation levels, although weak, is in line with previous evidence reporting correlations between mtDNA methylation and age of individuals [24,27,51,52]. In two previous reports, we observed an inverse correlation between D-loop methylation levels and age at sampling in cohorts including healthy subjects and patients with Parkinson’s disease [24,27]. Peripheral blood methylation levels of the *MT-RNR1* were positively associated with increasing age, and subjects with higher methylation levels showed a higher mortality risk than those with lower methylation levels [51]. On the other hand, a subsequent study identified two CpG sites within the *MT-RNR1* gene that were inversely correlated with the subject’s age [52]. Altered levels of mtDNA methylation have also been associated with the senescence process [53,54,55,56]. These observations are of particular interest with respect to AD, considering that its most common risk factor is advancing age and that mitochondria contribute to specific aspects of the aging process including cellular senescence and the decline in age-dependent cellular activity [57]. However, we are aware that the correlation between D-loop methylation and age at sampling observed in the current study is weak, and its potential biological and clinical significance could not be very relevant to our study population.

In conclusion, the results of the present study suggest that mitochondrial methylation of the D-loop is associated with different stages of AD, and, more interestingly, these methylation changes are identifiable in the peripheral blood, thus potentially providing biomarkers of the disease. Although we are aware that few individuals diagnosed with MCI or AD in the early stages of the disease were included in the study, and that current findings should be considered as preliminary results of a pilot study, statistical power suggested that the study was adequately powered to detect either small or robust D-loop methylation differences among the groups studied. Future studies conducted in a larger cohort of individuals with well-characterized MCI and early AD followed for a long time could confirm whether D-loop methylation levels are dynamically regulated during AD progression as observed in animal studies and whether they could indeed be considered valid peripheral biomarkers of the pathogenesis of AD.

## 4. Materials and Methods

### 4.1. Study Population

The study population includes a total of 207 individuals (Table 3), including 102 patients in the spectrum of Alzheimer’s disease (AD) and 105 age- and sex-matched neurologically healthy controls. According to their clinical dementia rating (CDR) score, AD patients were classified as mild cognitive impairment (MCI) patients with questionable or very mild dementia (CDR = 0.5; *n* = 14), early-stage AD patients with mild dementia (CDR = 1; *n* = 18), and late-stage AD patients with moderate to severe dementia (CDR ≥ 2; *n* = 70).

D-loop methylation data of control subjects and of AD patients at advanced stages of disease were derived from a previously described dataset [29]. All the subjects underwent a rigorous neurological examination at the Neurological Clinic of the Pisa University, and diagnosis of probable AD was performed according to DSM-IV [58] and NINCDS-ADRDA criteria [59]. All AD patients at advanced stages of disease had an MMSE score ranging from 10 to 20 and a CDR scale ≥ 2. Diagnosis of AD patients in the early stages of the disease and of MCI patients was performed according to NIA-AA-2011 and IWG-2 criteria [3,59,60] at the Neurological Clinic of Pisa University. All patients underwent a routine diagnostic workup with a physical and neurological examination, complete neuropsychological evaluation including mini-mental state examination (MMSE), brain magnetic resonance imaging (MRI), and brain positron emission tomoscintigraphy with (18F) fluorodeoxyglucose. They also underwent a biological characterization, including cerebrospinal fluid (CSF) measurement of beta-amyloid fraction 1–42 (Aβ1-42), and both total and phosphorylated at Thr181 site Tau protein levels (t-Tau and p-Tau181, respectively), as reported elsewhere [61]. All MCI and early AD subjects were positive for CSF biomarkers (*n* = 14, cutoff levels >275 pg/mL for t-tau, >60 pg/mL for p-tau, and <600 pg/mL for Aβ1-42) and/or for cortical amyloid PET (*n* = 18). Five of the 14 MCI patients (35.7%) converted to AD within two years. Biological samples from these subjects were only available at baseline, so they were still considered MCI for statistical analyses. As normal controls, we recruited healthy volunteer subjects matched to patients for age and sex, as well as for ethnicity and geographic origin (both patients and control individuals were Italian Caucasians, residents in northern Tuscany). Cognitive functions and family history of dementia were ascertained in controls, including only healthy subjects with no presence of cognitive impairment and with no relatives who developed AD or other dementias. All the included participants were not taking drugs, supplements, or substances known or suspected to interfere with DNA methylation.

### 4.2. D-Loop Methylation Analysis

Mitochondrial D-loop region methylation levels were assessed by means of the methylation-sensitive high resolution melting (MS-HRM) technique as reported elsewhere [29]. An aliquot of blood was collected from each subject in EDTA tubes and stored at –20 °C until assayed. DNA extraction was performed using the QIAmp DNA blood Mini Kit (Qiagen, Milan, Italy, Catalog N° 51106) following the manufacturer’s protocol. The extracted DNA was quantified using a NanoDrop ND 200c spectrophotometer (NanoDrop Thermo scientific, Wilmington, DE, USA). Two hundred ng of extracted DNA were treated with sodium bisulfite (Qiagen, Milan, Italy, Catalog N° 59104) in order to convert all unmethylated cytosines into uracil. A CFX96 Real-Time PCR detection system (Bio-Rad, Milan, Italy) was used to perform the MS-HRM. The following protocol was used: a first step at 95 °C for 12 min was followed by 50 cycles of 95 °C for 30 s, 56 °C for 45 s, and 72 °C for 30 s, followed by an HRM step of 95 °C for 10 s and 50 °C for 1 min, 65 °C for 15 s, and continuous acquisition to 95 °C at one acquisition per 0.2 °C. PCR reaction was performed in a final volume of 10 μL, containing 5 μL of master mix (Bio-Rad, Catalog N° 1725112, Milan, Italy), 10 pmol of each primer, and 10 ng of bisulfite-modified DNA template. Primers (Table 4) were designed by us using the software MethPrimer primer [62].

Standard DNA samples with known methylation levels (0, 12.5, 25, 50, 75 and 100%) were obtained by mixing fully methylated and fully unmethylated DNA (EpiTect methylated and unmethylated human control DNA, bisulfite converted, Qiagen, CatalogNo. 59695, Milan, Italy), and were included in each experiment in order to generate standard curves used to obtain the methylation ratio of each sample.

### 4.3. Statistical Analyses

Kolmogorov–Smirnov test was used to test the normality of D-loop methylation data. Since they were not normally distributed, natural logarithm transformation was performed before statistical analysis. Age at sampling and sex were compared among groups by means of one-way ANOVA and chi-square test, respectively. In order to investigate differences in D-loop methylation levels among groups, analysis of covariance (ANCOVA), including age at sampling, followed by a post hoc Bonferroni’s correction for multiple comparisons, was employed. Pearson correlation coefficient was used to evaluate the correlation between D-loop methylation levels and age at sampling. Partial correlations were calculated to examine the relationship between D-loop methylation levels and clinical and biochemical parameters, placing age as a control variable. The ability of D-loop methylation to distinguish MCI patients from the other groups was evaluated by performing receiver operating characteristic (ROC) curve analysis and estimating the area under the curve (AUC) value calculated with 95% confidence intervals (CIs). Statistical analyses were performed with STATGRAPHICS 5.1 (Statgraphics Technologies, Inc., The Plains, VA, USA) plus software package for Windows, the MedCalc statistical software version 12.5 (MedCalc Software, Ostend, Belgium), and IBM SPSS version 28.0 (IBM Corp., Armonk, NY, USA). Figures were obtained with GraphPad PRISM version 6.01 (GraphPad Software, San Diego, CA, USA). The statistical power of the study was calculated with G*Power, version 3.1.9.7 (Heinrich Heine University Düsseldorf, Germany). A post hoc analysis based on MS-HRM data observed in our population revealed that the study had a power greater than 80% to detect mean D-loop methylation differences of about 1% or higher among groups.

## Figures and Tables

**Table 1:** Mean D-loop methylation levels in control subjects, in MCI patients, and in patients with AD in the early and advanced stages of the disease.

	Control Subjects	MCI Patients	Early Stages AD	Advanced Stages AD
D-loop methylation levels (%, mean ± SD)	3.80 ± 3.73	6.77 ± 3.74	3.37 ± 4.51	2.12 ± 2.53

**Table 2:** Partial correlations controlling for age between D-loop methylation levels and clinical and biochemical parameters.

	MMSE	CSF t-Tau	CSF p-Tau	CSF Aβ1-42
D-loop methylation levels	r = 0.05; *p* = 0.76	r = −0.53; *p* = 0.062	r = −0.64; *p* = 0.025	r = −0.39 *p* = 0.18

**Table 3:** Demographic characteristics of the study population.

Characteristic	Controls (*n* = 105)	MCI (*n* = 14)	Early Stages AD (*n* = 18)	Advanced Stages AD (*n* = 70)	Statistics
Age (mean ± SD)	72.3 ± 6.3	70.1 ± 6.3	70.6 ± 6.4	72.4 ± 4.5	*p* = 0.37 ^a^
Gender (F/M)	61/44	6/8	14/4	48/22	*p* = 0.11 ^b^
MMSE (mean ± SD)	-	26.2 ± 4.0	18.1 ± 5.9	<20	*p* = 0.0001
CSF t-tau ^c^ (pg/mL), (mean ± SD)	-	595.7 ± 200.4	674.3 ± 554.9	-	*p =* 0.73 ^a^
CSF p-tau ^c^ (pg/mL), (mean ± SD)	-	73.7 ± 8.5	81.8 ± 42.8	-	*p =* 0.63 ^a^
CSF Aβ_1-42_^c^ (pg/mL), (mean ± SD)	-	486.1 ± 101.6	610.7 ± 157.0	-	*p =* 0.10 ^a^

^a^ One-way ANOVA; ^b^ chi-square test; ^c^ data available for 7 MCI subjects and 7 early-stage AD.

**Table 4:** Main characteristics of the primers used to study D-loop methylation levels. Forward and reverse primers nucleotide sequences, amplicon size (AS), number of CpG sites analysed, and accession number (AN) with nucleotide positions (NP) of the region analysed are reported.

	Primer Forward	Primer Reverse	AS	Number of CpGs	AN and NP
D-loop region	5′GGAGTTTTTTATGTATTTGGTATTTT-3′	5′ACAAACATTCAATTATTATTATTATATCCT-3′	222 bp	10	J01415.2 (35–256)

## Data Availability

The datasets generated and/or analyzed during the current study are available from the corresponding author on reasonable request.
